# Pulmonary aspergillosis and cryptococcosis as a complication of COVID-19

**DOI:** 10.1016/j.mmcr.2022.01.003

**Published:** 2022-01-06

**Authors:** Edward C. Traver, Melanie Malavé Sánchez

**Affiliations:** a University of Maryland Medical Center, Division of Infectious Diseases, Baltimore, 21201, USA; b University of Maryland School of Medicine, Division of Infectious Disease, Baltimore, 21201, USA

**Keywords:** SARS-CoV-2, COVID-19, Aspergillus, Cryptococcus, CAPA

## Abstract

Invasive fungal infections may complicate infection by SARS-CoV-2 and increase morbidity and mortality. A 59-year-old man with multiple medical comorbidities was transferred to our hospital for worsening hypoxic respiratory failure due to COVID-19 and received high-dose corticosteroids and 2 doses of cyclophosphamide. He was diagnosed with pulmonary aspergillosis and cryptococcosis by culture of a bronchoalveolar lavage sample. This patient's secondary infections were likely due to treatment with immunosuppressants, his comorbidities, and his prolonged critical illness.

## 1 Introduction

Like other viral pneumonias, Coronavirus disease 2019 (COVID-19) pneumonia, caused by SARS-CoV-2, is associated with secondary bacterial and fungal pneumonias. Immunomodulatory therapy, including corticosteroids and tocilizumab, has experimentally demonstrated benefit in COVID-19, but may cause more frequent or severe secondary infections. COVID-19-associated pulmonary aspergillosis (CAPA) is a well-recognized complication of COVID-19, although knowledge of the clinical implications is emerging. The frequency of other fungal infections in COVID-19 is poorly understood [1]. Cryptococcosis as a complication of COVID-19 has been rarely reported. We describe in detail the first case of secondary co-infection with pulmonary aspergillosis and cryptococcosis after COVID-19 [2].

## 2 Case

A 59-year-old man was admitted to a different hospital in Maryland, USA in January 2021 with bilateral pneumonia. He had a history of chronic obstructive pulmonary disease (COPD) managed with an inhaled corticosteroid and a long-acting beta-agonist, coronary artery disease with 3 drug-eluting stents, complete heart block with a pacemaker, heart failure with preserved ejection fraction, type 2 diabetes mellitus not requiring insulin treatment, obesity, obstructive sleep apnea, cirrhosis due to hepatitis C virus infection status post treatment with sustained virologic response, and remote injection drug use. It is unknown if he was previously colonized with *Aspergillus* species. Six weeks prior to this admission, he was hospitalized with respiratory failure, likely related to COPD and heart failure. He required endotracheal intubation and was diagnosed with *Klebsiella pneumoniae* ventilator-associated pneumonia and diffuse alveolar hemorrhage. He was extubated after 12 days, completed 10 days of antibiotics, and received high-dose corticosteroids followed by a taper for a total of 20 days.

**Fig. 1:**
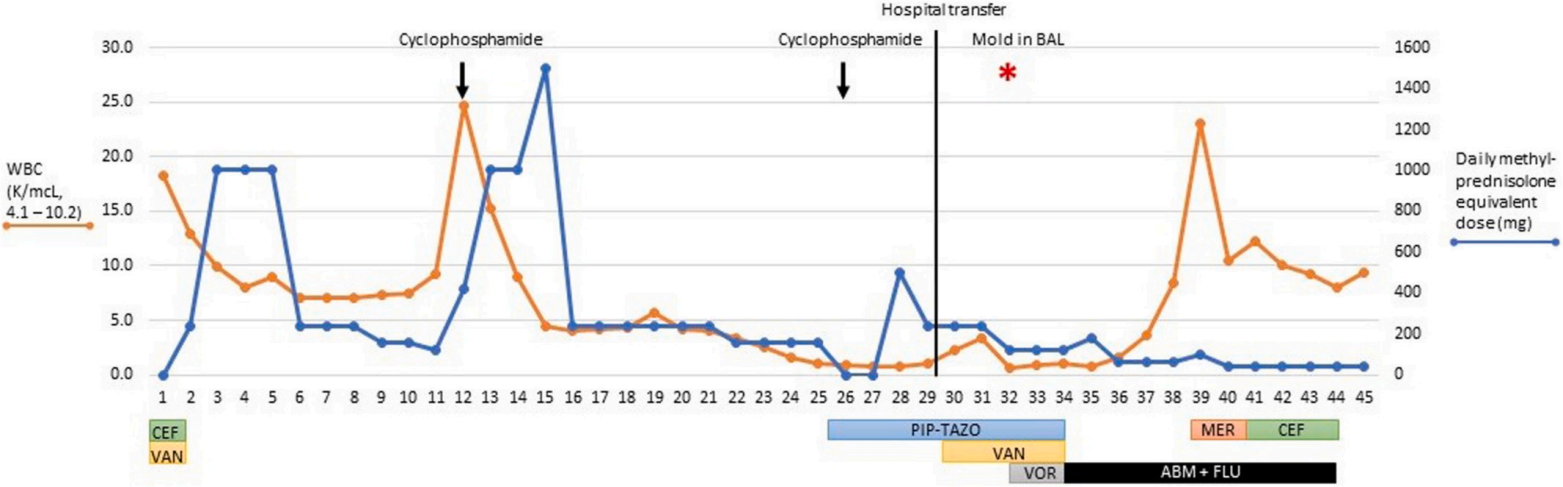
Timeline of hospital course. The patient received daily corticosteroids (blue line, methylprednisolone equivalent dose is [hydrocortisone dose (mg)/5]), and 2 doses of cyclophosphamide (black arrows). The peripheral WBC count (orange line) decreased after the cyclophosphamide doses. The patient was also treated with multiple antibacterial and antifungal drugs, as noted by the colored bars below the graph. AMB, amphotericin. BAL, bronchoalveolar lavage. CEF, cefepime. FLU, flucytosine. MER, meropenem. PIP-TAZO, piperacillin-tazobactam. VAN, vancomycin. VOR, voriconazole. WBC, white blood cells.

On day 1 of the current hospitalization, SARS-CoV-2 was detected by nucleic acid amplification, and he was treated with vancomycin and cefepime due to concern for concomitant bacterial infection. On hospital day 2, he was treated with methylprednisolone 60 mg IV every 6 hour, which was increased to 250 mg IV every 6 hours on day 3. Methylprednisolone was continued at varying doses through day 39. His hospital course and steroid dosing is summarized in Fig. 1. He initially required bi-level non-invasive ventilation but was weaned to high-flow supplemental oxygen via nasal cannula and transferred out of the intensive care unit. On hospital day 11, he developed worsened respiratory failure and was endotracheally intubated and mechanically ventilated. Due to concerns for both fibrotic lung disease and diffuse alveolar hemorrhage, the patient was treated with cyclophosphamide on hospital day 12. On hospital day 18, he was extubated but required low-flow supplemental oxygen. On day 26, he was treated for suspected secondary bacterial pneumonia with piperacillin-tazobactam due to worsening respiratory failure. On day 27, he received a second and final dose of cyclophosphamide. Over the following 2 days, he developed progressive bilateral diffuse pulmonary ground-glass opacities and was transferred to our hospital on day 30.

On arrival to our hospital, he required an immediate increase in oxygenation support with high-flow nasal cannula at 30 L per minute and a fraction of inspired oxygen of 70%. Methylprednisolone 60 mg IV every 6 hours, administered for refractory hypoxia, was slowly tapered. Piperacillin-tazobactam was continued for suspected bacterial pneumonia. Shortly after arrival, he was transferred to an intensive care unit and intubated for the second time. A bronchoscopy was performed and a bronchoalveolar lavage (BAL) was sent for culture. Intravenous vancomycin therapy was begun.

The white blood cell count decreased to a nadir of 600 per microliter on day 32. On day 33, the microbiological laboratory reported mold growing on the bacterial culture of the BAL. The patient was started on voriconazole. The next day, 1000 colony-forming units per mL (CFU/ml) of mold and 10,000 CFU/mL of yeast were identified on the bacterial BAL culture plate. The mold was identified as *Aspergillus fumigatus* by colony and sporulation structure morphology. The yeast was analyzed by matrix-assisted laser desorption/ionization and time-of-flight spectrometry and identified as *Cryptococcus neoformans*. Voriconazole was discontinued and the patient was treated with liposomal amphotericin B 3 mg/kg IV daily and 5-flucytosine 25 mg/kg by orogastric tube 4 times a day for severe pulmonary aspergillosis and cryptococcosis. On hospital day 34, a second BAL culture was obtained, which grew <1000 CFU/ml of *A. fumigatus* and 2000 CFU/ml of normal upper respiratory flora. On hospital day 35, a lumbar puncture revealed normal opening pressure and cryptococcal antigen was not detected in the cerebral spinal fluid. No tests for beta-D glucan or galactomannan were collected.

On day 39, the patient developed worsening hypoxia and septic shock. Taper of the methylprednisolone was completed and the hydrocortisone was started for refractory shock. The patient was diagnosed with pneumonia and was treated with meropenem, which was changed to cefepime 2 days later, when *Enterobacter cloacae* grew on an endotracheal aspirate culture. No fungi were identified on that culture. Methylprednisolone was changed to hydrocortisone 50 mg IV every 6 hours [equivalent to 40 mg of methylprednisolone per day [3]]. On day 43, the patient developed severe acute kidney injury. Due to the patient's stated wishes to avoid prolonged mechanical ventilation and renal replacement therapy, his surrogate decision makers withdrew life support. The patient died on day 45.

## 3 Discussion

We report a patient with severe COVID-19 complicated by pulmonary aspergillosis and cryptococcosis. He had a history of lung disease, diabetes, obesity, and recent admission for respiratory failure requiring intubation and corticosteroids. While hospitalized with COVID-19, he developed profound leukopenia after high doses of corticosteroids and two doses of cyclophosphamide. He also received multiple courses of broad-spectrum antibiotics. It was not clear if his clinical progression and death was a consequence of pulmonary aspergillosis and cryptococcosis. While there is emerging understanding of pulmonary aspergillosis in COVID-19, there are few reports of cryptococcosis.

The diagnosis of CAPA is challenging due to the uncertain significance of serologic and respiratory tract tests, but recent guidelines by the European Confederation for Medical Mycology and the International Society for Human and Animal Mycology provide criteria for possible, probable, and proven CAPA for clinical trials [4]. Our patient met criteria for probable pulmonary CAPA based on consistent radiographic infiltrates and a positive culture from the BAL. It is possible CAPA may have been diagnosed earlier with the use of fungal markers (beta-d-glucan and galactomannan from serum and galactomannan from respiratory samples), but these tests were not performed. At the first hospital, fungal pneumonia may not have been considered due to anchoring bias regarding the diagnoses of pulmonary fibrosis and alveolar hemorrhage. At our hospital, these tests are performed by a remote reference laboratory, limiting the real-time utility. Nevertheless, had the BAL culture not grown a pathogen, the tests may have been useful in this case and should be considered when evaluating for CAPA [4].

Distinguishing colonization and infection from aspergillosis in the setting of COVID-19 has been challenging. Detection of *Aspergillus* in patients with COVID-19 may reflect a wide spectrum of aspergillosis, from colonization to tracheobronchitis to angioinvasive pulmonary disease. For bacteria cultured from the respiratory tract, quantitative cultures of BAL specimens have used a threshold of 10,000 CFU/ml used to denote true pneumonia. It is unclear if quantification of fungal respiratory cultures provides clinically relevant information. Current CAPA diagnosis guidelines do not use quantitative thresholds of cultures to meet microbiologic criteria [4]. Our patient's *A. fumigatus* culture grew 1000 CFU/ml, which decreased to <1000 CFU/ml a few days later and was not cultured on the last respiratory sample. The *C. neoformans* was not cultured on subsequent cultures. The significance of these changes is unknown.

Evidence suggests that patients meeting criteria for CAPA have worse clinical outcomes. A prospective cohort study found higher 30-day mortality in patients admitted to the intensive care unit with CAPA compared to those without aspergillosis (44% v. 19%) [5]. It has been difficult to prove that aspergillus infection causes worse disease, rather than serves as a marker for severe COVID-19 or comorbid diseases. Another prospective cohort study of 135 patients with COVID-19 found higher mortality for patients with CAPA, and this effect was limited to those who did not receive antifungal therapy [6], suggesting a true pathogenic effect of aspergillosis. Increased mortality was also demonstrated by a multicenter study with mixed prospective and retrospective cohorts, which found lower intensive care unit survival among COVID-19 patients with proven, probable, or possible CAPA compared to those without CAPA [7]. Not all studies, however, had found increased mortality risk. A retrospective study of 396 mechanically ventilated patients with COVID-19 found that those with CAPA did not have increased mortality, but did have more severe disease, faster progression, slower improvement, and spent a longer time intubated and hospitalized [8]. A meta-analysis of 28 studies estimated a mortality rate of CAPA of 55% [9].

The incidence of CAPA varies among different studies and likely reflects the diagnostic criteria and the population examined. Incidence may vary over time, reflecting changes such as viral variants, patient vaccination, constraints on care delivery, frequency of mechanical ventilation, and use of immunosuppressants and antivirals. An analysis of autopsies of patients with COVID-19 found only 2% had proven invasive mold disease [10]. Incidence of CAPA based on clinical criteria has varied from 10% to 28% [5,6,8]; a meta-analysis found the incidence to be 10% with a 95% confidence interval of 8–13% [9]. The lower frequency of invasive disease on autopsy compared to clinical diagnosis suggests some of the clinically diagnosed cases reflect colonization or less severe disease.

It is likely that our patient's risk for fungal disease after COVID-19 was increased by his COPD, cirrhosis, mechanical ventilation, and treatment with cyclophosphamide and high-dose corticosteroids. CAPA has been associated with pre-existing lung disease [6] and mechanical ventilation [7]. Many studies have found increased risk of CAPA in patients treated with corticosteroids [6,8]. This association may partly reflect the use of corticosteroids in severe COVID-19 requiring mechanical ventilation. Other studies did not find an association of steroid use with CAPA [11] or all secondary infections [12], although they may have been underpowered for these findings. Tocilizumab is another immunosuppressant with benefit in severe COVID-19 and is also associated with CAPA [7], but our patient did not receive tocilizumab due to the prolonged duration of his respiratory failure after arrival to our hospital.

Antifungal prophylaxis against aspergillus has been shown to decrease invasive aspergillus infections among patients with severe iatrogenic immune suppression, such as those receiving chemotherapy or stem-cell transplants [13]. While studies have shown high rates of aspergillus colonization among people with COPD [14], it is not known if prophylaxis would be beneficial in this population during periods of immune suppression, such as high-dose corticosteroids, or in the setting of severe COVID-19.

This patient received cyclophosphamide prior to transfer to our hospital for alveolar hemorrhage and steroid-refractory pulmonary fibrosis. The impact of cyclophosphamide on risk of fungal diseases in COVID-19 is unknown. There are case reports of patients treated with cyclophosphamide for concomitant malignancy or autoimmune disease shortly before or after infection with SARS-CoV-2, but they do not describe secondary infections [15,16].

There is limited understanding of the pathogenesis, incidence, diagnosis, and risk factors for pulmonary cryptococcosis in COVID-19. In a prospective cohort of 135 patients with COVID-19, there were 17 cases of invasive yeast infection, but none were cryptococcosis [6]. We found 5 case reports of cryptococcosis complicating COVID-19. There are 3 reports of blood stream infection with *C. neoformans*, all in patients who received immunosuppressive medications, including corticosteroids [[17], [18], [19]]. All 3 patients died prior to discharge from the hospital.

Ghanem et al. reported a case of *C. neoformans* meningitis after treatment of COVID-19 with corticosteroids; the patient was discharged to a rehabilitation facility with a tracheostomy and gastrostomy [20]. Cafardi et al. reported a patient with severe COVID-19 pneumonia who developed acute respiratory failure requiring intubation; BAL culture grew *C.* neoformans [21]. The patient developed renal failure and died. Like our patient, these cases were caused by *C. neoformans*, had severe disease, and suffered poor outcomes.

Our patient's cryptococcosis is consistent with known characteristics in patients without COVID-19. Cryptococcosis is associated with use of corticosteroids in patients without human immunodeficiency virus infection, highlighting the role of iatrogenic immune suppression [22]. Lastly, cryptococcosis in the setting of immunosuppression is more often caused by *C. neoformans* than *C. gattii* [23].

This case highlights many important points. Fungal infections as a complication of COVID-19 are myriad and likely more common and variable than is currently known [1]. Benefits and harms of immunosuppression in patients with COVID-19, such as prolonged courses of corticosteroids and cyclophosphamide, must be carefully weighed, as they likely increase the risk of secondary fungal infections. The development of COVID-19-associated mucormycosis in India was also associated with use of corticosteroids [24]. In patients with refractory respiratory failure, establishing a diagnosis may help avoid unneeded treatment, such as cyclophosphamide for suspected alveolar hemorrhage. Future studies may clarify the role of antifungal prophylaxis in high-risk patients with COVID-19. Pulmonary cryptococcosis may be a rare complication of COVID-19, but the true incidence and implications are largely unknown.

## Ethical Form

Please note that this journal requires full disclosure of all sources of funding and potential conflicts of interest. The journal also requires a declaration that the author(s) have obtained written and signed consent to publish the case report from the patient or legal guardian(s).

The statements on funding, conflict of interest and consent need to be submitted via our Ethical Form that can be downloaded from the submission site www.ees.elsevier.com/mmcr. **Please note that your manuscript will not be considered for publication until the signed Ethical Form has been received.**

## Declaration of competing interest

There are none.
